# Exploiting the Role of Hypoxia-Inducible Factor 1 and Pseudohypoxia in the Myelodysplastic Syndrome Pathophysiology

**DOI:** 10.3390/ijms22084099

**Published:** 2021-04-15

**Authors:** Ioanna E. Stergiou, Konstantinos Kambas, Aikaterini Poulaki, Stavroula Giannouli, Theodora Katsila, Aglaia Dimitrakopoulou, Veroniki Vidali, Vasileios Mouchtouris, Ismini Kloukina, Evangelia Xingi, Stamatis N. Pagakis, Lesley Probert, George P. Patrinos, Konstantinos Ritis, Athanasios G. Tzioufas, Michael Voulgarelis

**Affiliations:** 1 Department of Pathophysiology, School of Medicine, National and Kapodistrian University of Athens, 11527 Athens, Greece; stergiouioanna@hotmail.com (I.E.S.); aikaterini.poulaki@gmail.com (A.P.); agtzi@med.uoa.gr (A.G.T.); 2 Laboratory of Molecular Genetics, Department of Immunology, Hellenic Pasteur Institute, 11521 Athens, Greece; kkampas@hotmail.com (K.K.); lesley.probert@gmail.com (L.P.); 3 Hematology Unit, Second Department of Internal Medicine, School of Medicine, National and Kapodistrian University of Athens, 11527 Athens, Greece; 4 Institute of Chemical Biology, National Hellenic Research Foundation, 11635 Athens, Greece; thkatsila@eie.gr; 5 Laboratory of Flow Cytometry, Immunology Department, Laiko Hospital of Athens, 11526 Athens, Greece; liladim1@hotmail.com; 6 Laboratory of Synthesis of Natural Products and Bioorganic Chemistry, Institute of Nanoscience and Nanotechnology, National Center for Scientific Research “Demokritos”, 15341 Athens, Greece; v.vidali@inn.demokritos.gr (V.V.); vmouchtouris@gmail.com (V.M.); 7 Center of Basic Research, Biomedical Research Foundation of the Academy of Athens, 11527 Athens, Greece; isminikloukina@gmail.com; 8 Light Microscopy Unit, Hellenic Pasteur Institute, 11521 Athens, Greece; evangeliaxingi@gmail.com; 9 Biological Imaging Unit, Biomedical Research Foundation of the Academy of Athens, 11527 Athens, Greece; spagakis@bioacademy.gr; 10 Department of Pharmacy, University of Patras, 26504 Patras, Greece; gpatrinos@upatras.gr; 11 First Department of Internal Medicine, University General Hospital of Alexandroupolis, Democritus University of Thrace, 68100 Alexandroupolis, Greece; rits2@otenet.gr

**Keywords:** myelodysplastic syndromes, hypoxia-inducible factor 1, pseudohypoxia, autophagy, mitophagy

## Abstract

Myelodysplastic syndromes (MDS) comprise a heterogeneous group of clonal hematopoietic stem (HSCs) and/or progenitor cells disorders. The established dependence of MDS progenitors on the hypoxic bone marrow (BM) microenvironment turned scientific interests to the transcription factor hypoxia-inducible factor 1 (HIF-1). HIF-1 facilitates quiescence maintenance and regulates differentiation by manipulating HSCs metabolism, being thus an appealing research target. Therefore, we examine the aberrant HIF-1 stabilization in BMs from MDS patients and controls (CTRLs). Using a nitroimidazole–indocyanine conjugate, we show that HIF-1 aberrant expression and transcription activity is oxygen independent, establishing the phenomenon of pseudohypoxia in MDS BM. Next, we examine mitochondrial quality and quantity along with levels of autophagy in the differentiating myeloid lineage isolated from fresh BM MDS and CTRL aspirates given that both phenomena are HIF-1 dependent. We show that the mitophagy of abnormal mitochondria and autophagic death are prominently featured in the MDS myeloid lineage, their severity increasing with intra-BM blast counts. Finally, we use in vitro cultured CD34+ HSCs isolated from fresh human BM aspirates to manipulate HIF-1 expression and examine its potential as a therapeutic target. We find that despite being cultured under 21% FiO2, HIF-1 remained aberrantly stable in all MDS cultures. Inhibition of the HIF-1α subunit had a variable beneficial effect in all <5%-intra-BM blasts-MDS, while it had no effect in CTRLs or in ≥5%-intra-BM blasts-MDS that uniformly died within 3 days of culture. We conclude that HIF-1 and pseudohypoxia are prominently featured in MDS pathobiology, and their manipulation has some potential in the therapeutics of benign MDS.

## 1. Introduction

Myelodysplastic syndromes (MDS) encompass a group of hematologic malignancies, which are characterized by clonal hematopoietic stem (HSCs) and/or hematopoietic progenitor cells (HPCs) expansion with defective differentiating potentials [1]. These clonal alterations dictate a heterogeneous clinical phenotype in which morphologic dysplasia, ineffective hematopoiesis with peripheral blood cytopenias, and increased risk of transformation to acute myeloid leukemia (AML) predominate [2]. The origins of the premalignant clones trace back to hematopoietic precursors that re-acquire self-renewal capacity with differentiation blockage [3], persist during treatment, and expand at times of relapse establishing not only the disease’s malignant potential, but also the grave prognosis MDS-related AML bears [4].

The identification and description of the biologic pathways leading to this transformation would divulge therapeutic targets and have thus concentrated global scientific interest. Although recurrent genetic aberrations including point mutations in epigenetic regulators, RNA splicing genes, transcription factors, and DNA response genes can be found in MDS HSCs/HPCs, a casual relation between them seems rather unlikely [5]. Up to 10–20% of normal individuals aged between 65 and 90 years present recurrent MDS-associated mutations, otherwise termed Clonal Hematopoiesis of Indeterminate Potential (CHIP), while the vast majority of the MDS clones show an impressive range of unique to each clone, genetic aberrations [6,7]. The universal economic impact of the supportive care offered to MDS patients, the grave prognosis of MDS-related AML, and the impressive genomic and phenotypic heterogeneity of the syndrome necessitate a rather holistic approach.

Based on their immature phenotype and the, at least initial, reliance of the dysplastic progenitors on the hypoxic bone marrow (BM) microenvironment, several unrelated research groups have underlined their similarities with healthy HSCs and have thus focused upon physiologic correspondence between them [8,9]. Healthy HSCs rely heavily on BM oxygen tension to retain their stemness, and they do so by altering their gene expression to fit a more glycolytic rather than mitochondrial metabolic profile [10,11]. One transcription factor lies centrally in this whole adaptation, hypoxia-inducible factor 1 (HIF-1) [12,13,14], which is a heterodimer of a constantly expressed beta (β) subunit and an oxygen-sensitive alpha (α) subunit [15]. Upon hypoxia, the α subunit is rescued from proteasomal degradation and the stable HIF-1 dimer upregulates the expression of genes coding for glucose transporters and enzymes involved in its extramitochondrial degradation, which is also known as the Warburg effect [16,17,18]. By positively regulating mitophagy, that is the autophagy of functional mitochondria, and by also optimizing the electron transport chain (ETC) of remaining organelles HIF-1 reduces the release of reactive oxygen species (ROS), ensuring quiescence [19,20,21]. Differentiation commitment is reflected on a metabolic switch from Warburg glycolysis to mitochondrial Tricarboxylic Acid Cycle (TCA) and OXidative PHOsphorylation (OXPHOS) marked by HIF-1α destabilization and rapid degradation [10,11,14]. The failure of such a transition could be accounted for the differentiation blockage, ineffective hematopoiesis, and malignant potential of the MDS clones putting HIF-1 centrally in the elusive MDS pathobiology.

In this study, we examine the aberrant stabilization of HIF-1α in human MDS BM-derived CD34+ and differentiating myeloid lineage using a major transcriptional HIF-1 target, DNA-damage-inducible transcript 4 (*DDIT4*) or regulated in development and DNA damage response 1 (*REDD1*) [22]. We show that HIF-1 stabilization is featured in all MDS subgroups, the phenomenon being oxygen independent and thus thereafter referred to as pseudohypoxia [23]. We further suggest an indocyanine (IDC) labeled nitroimidazole derivative (NIM) as a marker of the established pseudohypoxia both in isolated myeloid lineage and BM biopsies and suggest that the degree of NIM-IDC accumulation is directly associated with MDS severity, that is, with intra-BM blast percentage. Finally, we assess the permissive role of HIF-1 in MDS ineffective myelopoiesis and thus its potential as a therapeutic target. Using a functional HIF-1α inhibitor and *in vitro* cultures of human BM-derived CD34+ HSCs under myeloid priming, we show that HIF-1α inhibition has a moderate beneficial effect, both qualitative and quantitative, on the low-risk MDS cultures, while it does not suffice to rescue the culture from demise in MDS with excess blasts 1/2 experiments. We conclude that HIF-1 inhibition may be an appealing target to relieve neutropenia in some benign MDS patients, yet it still remains an epiphenomenon of a global BM microenvironmental interruption that necessitates extensive research.

## 2. Results

### 2.1. Human MDS CD34+ and Myeloid-Lineage Cells Display an Activated HIF-1 Profile with Increased REDD1 Protein Levels

**Figure 1:**
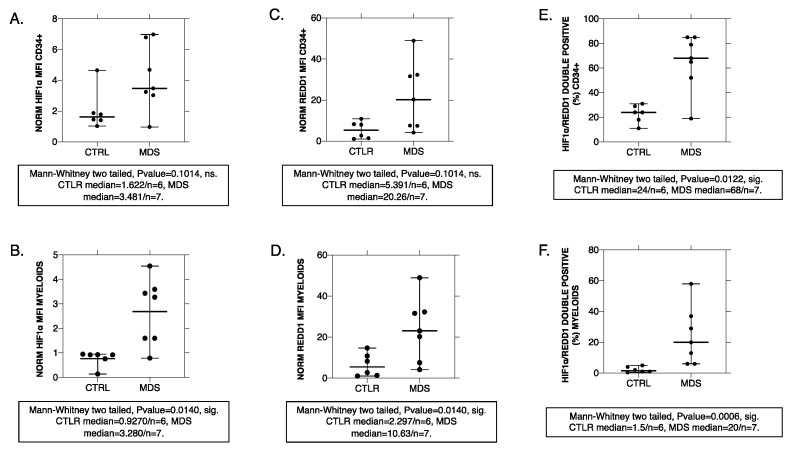
Hypoxia-inducible factor 1 (HIF-1α) and regulated in development and DNA damage response 1 (REDD1) protein kinetics in CD34+ and differentiating myeloid lineage from controls (CTRL, n = 6) and myelodysplastic syndromes (MDS, n = 7) patients. HIF-1α (**A**,**B**), REDD1 (**C**,**D**) expression as well as HIF-1α/REDD1 co-expression (**E**,**F**) were assessed through flow cytometry with subsequent statistical analysis to yield significance. Although great variability exists in the MDS group owing to and reflecting upon the syndromic nature of this clinical entity, a difference does exist between CTRLs and MDS even if significance fails to be established (**A**,**B**). No correlation was established with MDS severity; thus, all samples, namely both from <5% and ≥5% intra BM blast counts were assessed together. However, we do underline that peak HIF-1α and REDD1 median fluorescence intensity (MFI) values in myeloids, the differentiation stage at which HIF-1α levels and thus MFI ought to be physiologically declining, correspond to low-risk patients (**B**,**D**). In the CD34+ group, such a correlation was not observed (**A**,**C**). The phenomenon is also reflected upon the pattern of HIF-1α/REDD1 coexpression (**E**,**F**) where peak coexpression also corresponds to the same low-risk patients. The great variability in coexpression in the MDS group suggests a more complex nature of REDD1 upstream regulation aside form HIF-1 stability that is observed not only in the myeloid group (**F**) but also in the CD34+ HSCs (**E**). ns: non-significant, sig.: significant. Mann–Whitney *p*-value > 0.05 or <0.05 accordingly.

To investigate HIF-1 signaling in human myelodysplasia, we assessed mRNA and protein levels of HIF-1α and HIF-1 direct transcriptional target, *REDD1*, in BM samples of 16 previously untreated MDS (or seven for protein levels) patients and seven matched controls (or six for protein levels). *HIF1A* mRNA levels, by quantitative real-time PCR (qRT-PCR), did not differ between MDS patients and controls, while *REDD1* mRNA was significantly increased in MDS CD34+ cells and myeloid precursors (Supplementary Figure S1A). Moreover, protein expression levels assessed by flow cytometry in seven MDS patients (n = 4 MDS-multilineage dysplasia (MLD), n = 2 MDS-excess blasts 1 (EB1), n = 1 MDS-excess blasts 2 (EB2)) and six controls showed up-regulation of both HIF-1α and REDD1 peptides in MDS CD34+ and differentiating myeloid cells, yet with significance established only in the latter group (Figure 1A,B,D,E). This may lie upon the inherent HIF-1 positivity of CD34+ HSCs [14], which sequentially leads to REDD1 protein expression in health. When assessing the double positivity for HIF-1α and REDD1, significant differences are observed both in the CD34+ and the myeloid group (Figure 1C,F). The phenomenon implies alternative regulatory pathways leading to additional REDD1 transcriptional activation that are present even from the CD34+ HSCs stage in the control group that were further confirmed when assessing *REDD1* mRNA levels in control CD34 + cultures upon HIF-1α inhibition (Supplementary Figure S1B). Our results do show that HIF-1 is aberrantly active in MDS even from the CD34+ HSCs stage and that this is due to posttranscriptional HIF-1α peptide regulation. We also show that a HIF-1 transcriptional target, REDD1, is also aberrantly expressed and that this is partially and most likely predominantly due to HIF-1 dependent regulation establishing the functional HIF-1 transcriptional program. Of note, the great variability observed in protein expression levels of both CD34+ and myeloid groups is attributed to the polymorphic nature of the MDS pathophysiology and subsequent syndromic clinical phenotype (Figure 1A–F). Examining the MDS subgroups based on their severity (MDS-MLD, low severity with <5% blasts versus MDS-EB1 and MDS-EB2, high severity with ≥5%), no correlation was established between HIF-1α and REDD1 protein expression levels and the MDS subgroup analyzed. We should state though that peak HIF-1α and REDD1 protein expression in the differentiating myeloid cells were found in patients with MDS-MLD (Figure 1B,D), while this phenomenon was not observed in CD34+ positive cells. Peak HIF-1α/REDD1 protein coexpression levels corresponded to same patients with MDS-MLD (Figure 1E,F).

### 2.2. MDS BM Shows Increased Intracellular NIM-IDC Accumulation That Positively Correlates with MDS Severity

**Figure 2:**
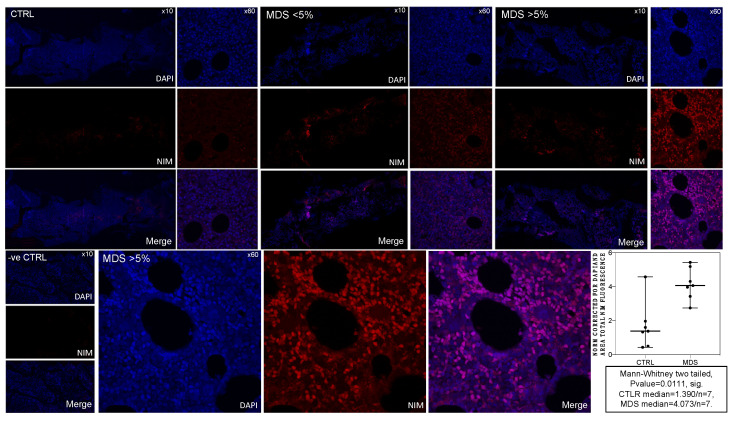
Bone marrow biopsy nitroimidazole derivative (NIM)-indocyanine (IDC) (Red) immunofluorescence from control (CTRL/n = 7) and MDS (n = 7) subjects. One representative CTRL, and two MDS along with the negative control (-ve CTRL) samples are presented. 4.6-diamidino-2-phenylindole (DAPI) (Blue) was used as a contrast stain given its very different excitation and emission spectrums with NIM-IDC. Both whole section tile scans (×10 and ×20) along with corresponding close-ups (×60) are shown. Close ups form the MDS ≥5 % intra BM blast counts are also shown enlarged to visualize the intracellular NIM-IDC staining. Colocalization of NIM-IDC with DAPI is attributed to up-regulated apoptosis and was corrected when calculating the NIM-IDC fluorescence along with differences in area section. For two out of seven CTRL and MDS samples, two sections from different depths within the biopsy specimen were microscopically observed to ensure method accuracy. We show statistically significant intracellular NIM-IDC immunofluorescence in MDS samples with its accumulation increasing directly with the severity of MDS. Of note, MDS severity is also visually directly associated with apoptotic burden, which is further analyzed in the main text. ns: non significant, sig.: significant. Mann–Whitney *p*-value > 0.05 or <0.05 accordingly.

To solidify our hypothesis that HIF-1α stabilization is the net effect of a problematic MDS BM micro- and intracellular environment, we examined BM respiration using a conventional hypoxic marker nitroimidazole derivative, 2-NIM, conjugated with the fluorescent dye indocyanine (IDC) (Supplementary Table S3). Whole BM samples were immersed into the liquid immediately after acquisition (see Materials and Methods). Confocal microscopy of BM biopsy from both controls (CTRLS/n = 7) and MDS (n = 3 MDS-EB1/2, n = 4 MDS-MLD) yielded statistically significant differences in NIM-IDC immunofluorescence between the two groups (Figure 2 lower right panel). To increase the validity of our results, we corrected NIM-IDC immunofluorescence for cellularity and area by dividing with 4.6-diamidino-2-phenylindole(DAPI) immunofluorescence. Thus, we also corrected for areas of DAPI/NIM colocalization (see Materials and Methods). We established statistically significant increased intracellular NIM-IDC accumulation in the MDS group (Figure 2 lower right panel). Moreover, corrected total NIM-IDC immunofluorescence directly correlated with MDS severity, that is, with intra-BM blast counts. The observed DAPI/NIM colocalization was also markedly increased with increasing MDS severity (Figure 2). According to morphologic features of cells where colocalization was evident, we suggest that these might correspond to apoptotic nuclei, yet given our lack of experimental data to support such an assumption, we further analyze our theory in the Discussion section. Increased NIM-IDC accumulation in the dysplastic BM is not attributed to increased intra BM CD34+ blasts. The sample with MLD (multilineage dysplasia) and 0% intra BM blasts showed markedly increased NIM-IDC accumulation compared to controls. Confocal close ups (×63/1.4 NA objective) and subsequent z stacks identify differentiating erythroid and myeloid lineage with cytoplasmic NIM-IDC concentrates (Figure 2 lower middle panels). We avoided using double-stain immunofluorescence in the NIM-IDC embedded BM specimens to reduce biased signals given the auto-fluorescent IDC properties. We conclude that at the cellular level in the MDS BM, pseudohypoxic conditions cause the accumulation of NIM-IDC, and we suggest that NIM-IDC dye can be used as a marker of pseudohypoxia observed in MDS yet with certain caveats.

### 2.3. Mitophagy and Autophagic Cell Death Are Prominently Featured in the MDS Myeloid Lineage; Their Severity Increasing with the Intra BM Blast Counts

**Figure 3:**
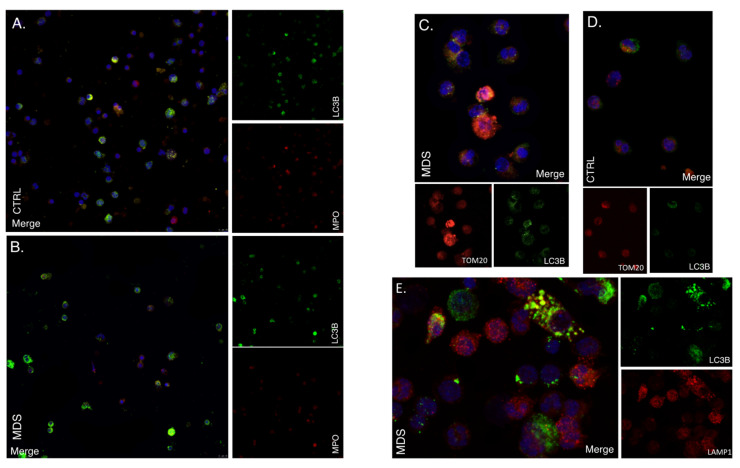
Autophagy is up-regulated in differentiating myeloid cells from MDS patients. (**A**,**B**) Autophagy levels in myeloid cells (high density layer) after density gradient separation, as assessed by LC3B immunofluorescence. Red: MPO, Green: LC3B, Blue: DAPI. Objective 63×/1.4NA. One representative out of four independent experiments is shown. (**C**,**D**) Active mitophagy in myeloid cells from MDS patients and CTRL as assessed by LC3B/TOMM20 immunofluorescence Red: TOMM20, Green: LC3B, Blue: DAPI. Objective 63×/1.4NA-representative close-up. One out of two independent experiments is shown. (**E**) Immunofluorescence of LC3B and LAMP-1 in myeloid cells of MDS patients. Objective 63×/1.4NA representative close-up. Red: LAMP1, Green: LC3B, Blue: DAPI. One out of three independent experiments is shown. LC3B: Microtubule-associated proteins 1A/1B light chain 3B, MPO: Myeloperoxidase, TOMM20: Translocase Of Outer Mitochondrial Membrane 20, LAMP 1: Lysosomal-associated membrane protein 1.

Hypoxia-induced autophagy is part of a general mechanism of cell adaptation that is controlled by HIF-1 [14,24,25] and would therefore be also activated even upon non-canonical HIF-1 stabilization [24]. Therefore, we studied the autophagic status of BM CD34+ and MPO+ cells from five MDS patients and three controls [25]. Immunofluorescence for LC3B (Microtubule-Associated Protein Light Chain 3B) showed enhanced autophagosome formation, namely increased punctuated signals in MDS MPO+ cells, that is in the differentiating myeloid lineage (Figure 3A,B), while the phenomenon was virtually absent from CD34+ HSCs (Supplementary Figure S2). LC3B/TOMM20 (Translocase of Outer Mitochondrial Membrane 20) colocalization in the differentiating myeloid lineage of MDS samples placed mitochondria inside the LC3B+ autophagosomal membrane while LC3B/LAMP1 (Lysosomal Associated Membrane Protein 1) colocalization established autophagy completion with autophagosome–lysosome fusion (Figure 3C–E accordingly, Supplementary Figure S5).

**Figure 4:**
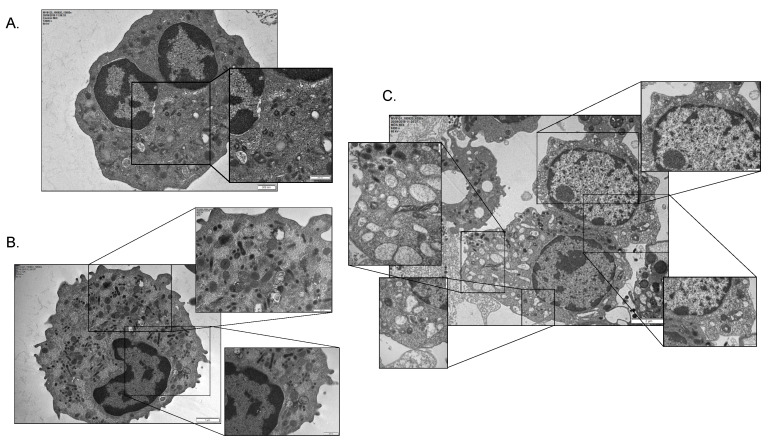
Electron microscopy of the differentiating myeloid lineage following whole bone marrow aspirate ficoll bilayer separation (high-density layer) from MDS patients (n = 4) and controls (n = 2). (**A**) Control, (**B**) MDS (<5% blasts), (**C**) MDS (≥5% blasts). (**A**) Mature healthy neutrophil with numerous small healthy mitochondria (outlined in the black panel). (**B**) Mature myeloid lineage with complete mitochondrial absence while with the presence of double membrane autophagosomes at variable maturation stages. (**C**) Markedly atypical maturing myeloid lineage without granules with nuclear immaturity. An abundance of large, elongated mitochondria and numerous maturing autophagosomes, some with engulfed mitochondria, is also evident. Scale bars and magnifications are shown on the upper left corner of each picture.

Next, the myeloid lineage of four MDS patients (n = 2 MDS-MLD, n = 1 MDS-EB1, n = 1 MDS-EB2) and two controls was examined under electron microscopy (Figure 4). Abundance of autophagic vacuoles, including double-membrane autophagosomes with engulfed mitochondria, confirmed the increased autophagic flux with active mitophagy in the MDS myeloid lineage (Figure 4B,C) [26]. Such features were absent in controls (Figure 4A). Importantly, when observing the MDS myeloid lineage, we came across interesting changes in their mitochondrial content. While samples from the benign, <5% intra BM blast group showed complete absence of mitochondria along with active mitophagy (Figure 4B), its malignant counterpart with ≥5% blasts presented with markedly disturbed mitochondrial morphology (Figure 4C and Supplementary Figures S3 and S4). Apart from an overwhelming amount of autophagic flux, high-risk myeloids possess elongated, abnormal, and even enormous for their differentiation stage mitochondria (Figure 4C and Supplementary Figure S4). The mitochondrial morphology of these cells raises concern that the aberrant HIF-1α stability observed at least in this group is indeed an epiphenomenon of the pseudohypoxia conferred by disturbed respiratory function.

Subsequently, the overwhelming autophagic flux leads the MDS differentiating myeloids to death. Nuclear membrane convolution and shrinkage, focal concavity of the nuclear surface, focal ballooning of the perinuclear space, numerous autophagosomes and autolysosomes, which along with a dilated and fragmented endoplasmic reticulum and swollen, electron-dense mitochondria with abnormal internal structure essentially translate to a distinctive type of autophagic cellular death called autosis (Figure 4C and Supplementary Figure S4). Classical features of apoptosis were variably present only in the low-risk MDS group. We conclude that increased autophagic flux in general and mitophagy specifically are prominently featured in the MDS differentiating myeloids. We show that their levels vary greatly with MDS severity/risk and that they are directly associated with intra BM blast counts. We also propose autosis as the main mechanism driving ineffective myelopoiesis in malignant MDS subtypes, while a mixed apoptotic-autotic pattern applies better to their benign counterparts. To this end, aberrant HIF-1 stabilization can be secondarily featured in the pseudohypoxic intracellular microenvironment of a greatly disturbed mitochondrial function.

### 2.4. HIF-1α Inhibition Enhances the Proliferative and Differentiating Capacity of MDS BM CD34+ Cells

**Figure 5:**
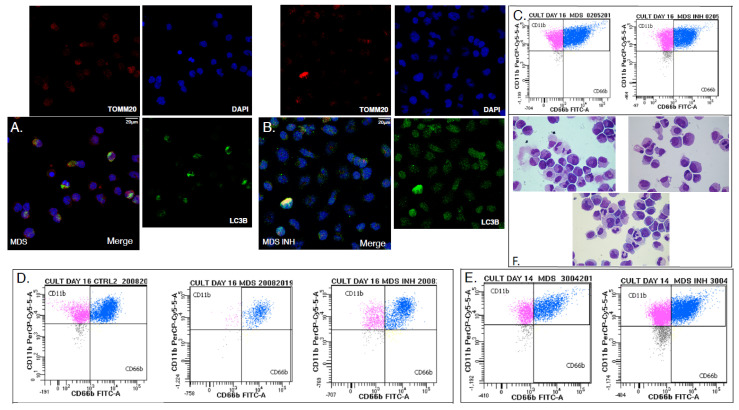
Effects of HIF-1α inhibition on human CD34+ in vitro cultures primed toward myeloid differentiation. (**A**,**B**) Assessment of differentiation, mitochondrial content, and autophagy through immunofluorescence for DAPI (blue), TOMM20 (red), and LC3b (green), objective 63×/1.4NA. (**A**) MDS, (**B**) MDS plus HIF-1α inhibitor culture day 16. (**C**) Flow cytometry for differentiation markers CD66b/CD11b and photon microscopy of culture cytospin showing differentiation improvement in one out of three presented cultures. (**D**,**E**) Flow cytometry for differentiation markers CD66b/CD11b of two independent experiments on days 16 (**D**) and 14 (**E**) show marked quantitative and qualitative improvement. (**F**) Photon microscopy of May-Gruenwald/Giemsa stained cytospin from the (**D**) culture on day 16 showing numerical expansion and differentiation improvement. Objective (×100). Upper left: Control, Upper right: MDS, Lower center: MDS+INH. All experiments were performed in CD34+ HSCs from patients suffering from MDS with <5% intra bone marrow blasts. One representative control is shown. Each MDS culture corresponds with one or more control cultures. LC3B: Microtubule-associated proteins 1A/1B light chain 3B, MPO: Myeloperoxidase, TOMM20: Translocase of Outer Mitochondrial Membrane.

To examine causality between the oxygen-independent HIF-1 activity and the MDS related hematopoietic failure, we used a functional HIF-1α inhibitor in normoxic *in vitro* cultures of human MDS BM-derived CD34+ cells primed toward myeloid differentiation. A total of six experiments were performed. As expected, the three CD34+ cultures from MDS with excess blasts 1/2 did not flourish either with or without HIF-1α inhibition. On the other hand, all CD34+ cultures from <5% intra BM blast count samples reacted variably to HIF-1α inhibition, all of them uniformly benefiting from it (Figure 5). On culture day 16 (or 14), we evaluated the differentiation stage and morphology using optical microscopy and flow cytometry for CD11b and CD66b, both of them common myeloid markers (Figure 5C–E) [27]. Out of three low-risk cultures, one showed marked improvement in cellularity and differentiation (Figure 5D,F), one showed moderate increase in numbers of mature forms (Figure 5E), and one showed only mild improvement in differentiation status (Figure 5C). Importantly, culture reaction to HIF-1α inhibition inversely correlated with culture behavior without it. Successful HIF-1α inhibition was further confirmed by reduced *REDD1* mRNA levels assessed with qRT-PCR (Supplementary Figure S1B). As expected, HIF-1α inhibition in control cultures did not significantly change *REDD*1 mRNA levels owing to HIF-1 independent, mostly stress-induced *REDD1* regulation in normality.

**Figure 6:**
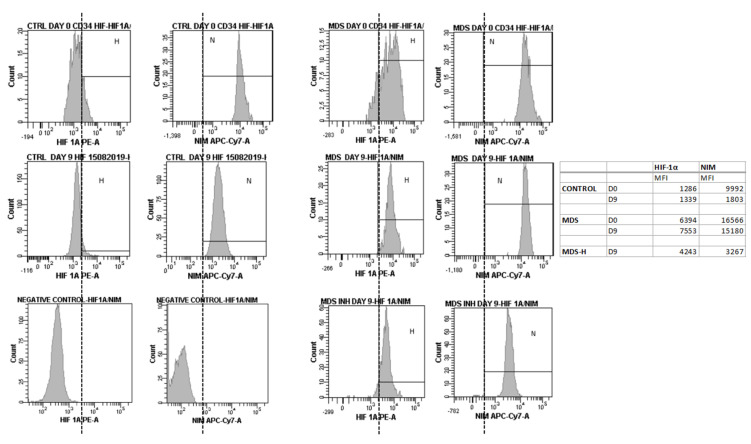
HIF-1α and NIM median fluorescence intensity (MFI) plots, assessed by flow cytometry, in differentiating control and MDS CD34+ cells cultured in vitro under normoxic conditions. Control cells exhibit low HIF-1α expression during differentiation, with a rather stable HIF-1α MFI (d0: 1286, d9: 1339). MDS cells maintain higher levels of HIF-1α expression during differentiation, even under normoxic conditions, with higher HIF1α MFI levels compared to controls and a trend of increase in HIF-1α MFI from d0 to d9 (d0: 6394, d9: 7553). NIM MFI tends to decrease during the normoxic culture of CD34+ control cells (d0: 9992, d9: 1803), while it exhibits higher levels that remain increased during the normoxic culture of MDS cells (d0:16566, d9: 15180). HIF-1α inhibition in MDS CD34+ cell culture decreases the levels of NIM expression on d9 (NIM MFI 3267 vs. 15180 without inhibition). Due to NIM-IDC autofluorescence, different samples from each culture were used to assess HIF-1α and NIM-IDC fluorescence each day.

HIF-1α inhibition also improved the autophagic status of the low-risk MDS differentiating myeloid cells (Figure 5A,B). Immunofluorescent for TOMM20 and LC3B on day 16 (or 14) showed that MDS cells treated with the inhibitor showed decreased but not diminished mitophagy compared to their untreated counterparts. Mitochondrial content assessed visually through TOMM20 immunofluorescence did not differ between the two groups. Of note, HIF-1α inhibition did not suffice to rescue any culture from neither high nor low-risk from demise. It did improve myelopoiesis, almost leading to normalization in cultures that were already viable even without the inhibitor. We suggest that although HIF-1 may largely contribute to ineffective myelopoiesis observed in MDS, its contribution varies with the MDS severity, while its therapeutic potentials are limited to a selected group of benign prognosis patients with <5% intra BM blasts MDS and yet ill-defined characteristics. Indeed, in those cultures that were benefited by HIF-1α inhibition, flow cytometric assessment of NIM-IDC accumulation yielded matching kinetics establishing the pseudohypoxic nature of the dye accumulation in MDS (Figure 6).

## 3. Discussion

The mechanisms underlying HIF-1 stabilization in the MDS BM remain essentially elusive. The oxygen sensing α subunit of the heterodimer was found to be uniformly increased in the MDS BM myeloid lineage despite adequate oxygen perfusion. Given that even culturing MDS CD34+ cells in 21% FiO_2_, which is an oxygen supply way over that of both the control and MDS BM, did not suffice to degrade HIF-1α (Figure 6), we strongly argue toward a non-canonical pathway of intracellular dysfunction driving the over-tuned HIF-1 activity and therefore will further discuss our theories as future experimental and subsequently therapeutic targets.

Upon normal conditions, when O_2_ is scarce, “anaerobic respiration” results in excessive free electron release, NIM reduction, and intracellular retention, making it a marker of inadequate O_2_ perfusion [28]. However, a defective mitochondrial electron transport chain would also result in excess free electron release and would also result in NIM reduction and retention, establishing the phenomenon of pseudohypoxia [23]. Subsequent intracellular retention requires binding of the reduced molecule with glutathione (GSH) and sequential GSH oxidation to oxidized glutathione (GSSG) [29]. The depletion of GSH has been accounted for NIM effects against several solid tumors [30]. In our case, increased NIM-IDC was observed with increasing MDS severity, and it did not at least morphologically accumulate in blast cells. Moreover, the absence of NIM-IDC accumulation in BM biopsies of control subjects obtained under the very same circumstances as those of MDS patients implies an oxygen-independent pathology (Figure 2). Given that the theory of hypoxic niches has proved rather obsolete and that both CD34+ HSCs and differentiating myeloid cell reside under uniform perfusion conditions [14,31,32], we can safely assume that the differential incorporation of NIM-IDC is in both control and MDS BMs based upon cell conditions intrinsic to each. We also observed increased DAPI/NIM-IDC colocalization directly correlating with MDS intra BM blast percentage. Colocalized areas are morphologically compatible with apoptotic nuclei, and given that the established increased apoptosis is a defining feature of MDS pathobiology [33], despite lacking adequate experimental data, we suggest that these areas correspond to apoptotic cells indeed. Indubitably, our assumption merits further investigation to be confirmed or dismissed. It is possible that segmentation of the nuclear membrane during the apoptotic process allows for the retained NIM-IDC to enter the nucleus (Figure 2 lower middle panels). In accordance with this hypothesis, our team has already established the reliance of differentiating cells of high-risk MDS on reduced GSH for survival and redox balance [34]. NIM-IDC is known to be retained intracellularly through binding with GSH and thus causing its depletion [28,31]. Given that maintenance of the redox balance, namely an increased GSH/GSSG ratio, is vital for survival in MDS myeloids, especially those from high-risk subgroups, we propose that DAPI/NIM-IDC colocalization areas refer to apoptotic nuclei of cells depleted from GSH. Under these circumstances, internalized dye entered the nucleus during the apoptotic nuclear membrane permeabilization and thus was visualized as DAPI/NIM-IDC colocalization. In such a case, corrected normalized NIM-IDC immunofluorescence largely underestimated true NIM-IDC fluorescence. Unfortunately, we could not use other fluorescent markers to establish our theory given the auto-fluorescent properties of the dye used.

While both an aberrant HIF-1α stabilization and NIM-IDC accumulation come in agreement with the pseudohypoxic theory, whether this state is due to a primary HIF-1α stabilization event or whether the latter is itself an epiphenomenon of an unknown etiology is still under question. In other words, is excessive mitophagy due to HIF-1 activation through a yet unidentified pathway a primary event, or does it lie upon intrinsic defects in mitochondrial respiration for instance, leading to HIF-1 stability and establishing the pseudohypoxic state? The answer may lie upon mitochondrial morphology itself. Mitochondrial morphology reflects largely mitochondrial function and kinetics [32]. Fission and fusion regulate mitochondrial mass by allowing the autophagic degradation of smaller organelles with defective complexes, while large elongated ones are rescued from degradation even after being encircled in autophagosomes inhibiting lysosome fusion [35,36]. Moreover, mitochondrial kinetics allow for ETC optimization of the surviving organelles by renewing their complexes. Notwithstanding, mitophagy that is so prominently featured in our experiments further regulates the quality of intracellular respiration [37]. The extremely disturbed mitochondrial morphology we observed in the myeloid lineage of MDS-ΕΒ1/2 denotes a secondary pseudohypoxic phenotype caused by faulty respiration and secondary HIF-1 stabilization. In fact, the elongation of the organelles in these cells is considered to be a compensatory phenomenon (Figure 4C). Our team has proved that disturbed mitochondrial function in both low and high intra BM blast percentage MDS by means of an aberrant metabolome [34]. Results of the present study come to confirm our data with the benign MDS subtypes suffering marked degradation of their dysfunctional mitochondria, leading to pseudohypoxia and HIF-1 stabilization while as disease progresses, genetic or epigenetic pressure leads to compensatory changes in mitochondrial function, and kinetics that remain faulty cause HIF-1 stability but are also rescued from degradation. Cultures we performed establish this notion (Figure 5 and Figure 6).

HIF-1 signaling has been shown to induce autophagy either through the mammalian target of rapamycin (mTOR)-dependent HIF-1/REDD1 axis or through the mTOR independent BNIP3–Beclin–Bcl2 pathway [38,39]. Autophagy has multiple roles in hematopoietic stem/progenitor cell (HSPC) fate and differentiation [40]. Mitophagy reduces mitochondrial mass and the resultant ROS production, thus protecting HSCs from DNA damage and ensuring their quiescence [21]. In addition, during terminal neutrophil differentiation, autophagy provides energy through fatty acid oxidation by degrading lipid droplets [41]. Therefore, it is essential for HIF-1 to be degraded in order for differentiation to ensue, which explains the kinetics and morphology improvement in cultures that benefited from MDS inhibition and establishes our suggestion that its aberrant activity is secondary to a wider disturbance.

Conversely, HIF-1 mediated aberrant propagation of autophagy results in cellular death through consumption of the whole endoplasmic reticulum lipid bilayer in the process of double membrane autophagosome formation [42,43]. A novel form of Na^+^, K^+^, ATPase regulated non-apoptotic cell death, termed “autosis”, has recently been identified, which among other stimuli can be induced by hypoxic signaling [43]. Our study demonstrated increased autophagic activity in MDS myeloid cells, specifically mitochondria (Figure 4 and Supplementary Figure S4). Electron microscopy featured the morphologic criteria of autosis, which is highly suggestive of the dominant role of this phenomenon in MDS-associated cytopenias more prominently in the high intra BM blast count subgroup (Figure 4C and Supplementary Figure S4), while death in the low blast count group was mostly apoptotic compatible with energy depletion. Following HIF-1 stabilization, mitophagy ensues as mentioned, gripping the now differentiating myeloids to this deadly cycle either through overwhelming autophagy, autosis, or through apoptosis due to energy and redox depletion.

While novelty in the field of MDS research has been vividly circling around HIF-1 and managed to ascribe its contribution or even necessity in the establishment of the dyshaematopoietic phenomena [44], a solely human model has yet to be studied along with its therapeutic potential. Our in vitro experiments do point out that while HIF-1 may have a permissive role on dyshaematopoiesis and specifically dysmyeloipoiesis, its inhibition certainly does not suffice to rescue all MDS CD34+ myeloid primed cultures (Figure 5). All cultures from MDS-EB1/2 patients failed to even initially expand, underlining the dependence of those CD34+ cells on the BM microenvironment and confirming the epiphenomenal nature of HIF-1α stabilization. Results in low-risk cultures were more variable and in general optimistic, unraveling a possible therapeutic role for HIF-1 inhibition in MDS associated neutropenia.

The different effect of HIF-1α inhibition in CD34+ myeloid primed cultures from low and high-risk MDS may lie in inherent differences in major cell signaling pathways. The phosphoinositide 3-kinase (PI3K)–mTOR axis is a major pathway involved in promoting HIF-1α transcription and translation, regardless of oxygen levels [45]. The PI3K–Akt–mTOR pathway is among one of the intracellular pathways aberrantly upregulated in AML, and its activation seems important in leukemogenesis, since it contributes to the regulation of cellular metabolism by several mechanisms, ensuring energy required for cell proliferation [46,47]. It has been shown that patients with high-risk MDS frequently show an activation of Akt compared with both low-risk MDS patients and healthy donors [48]. Activation of the Akt/mTOR pathway is critical for cell survival and proliferation in high-risk MDS, with studies showing that mTOR inhibition leads to a significant increase in apoptosis of isolated CD33+ bone marrow cells of high-risk MDS patients, which is an effect not observed in low-risk MDS [49]. Although HIF-1α is a positively regulated down-stream target of the PI3K/Akt/mTOR axis, HIF-1α inhibition alone could not suffice to offset the effects of an extremely enhanced PI3K/Akt/mTOR signaling with multiple other downstream targets, thus explaining the expansion failure observed in our cultured CD34+ cells of high-risk MDS patients. However, inhibiting HIF-1α in low-risk MDS CD34+, where the PI3K/Akt/mTOR signaling is dampened, seems to be effective in restoring myelopoiesis at least to some degree.

Phoshoinositide–Phospholipase C (PI-PLC) beta1 is a key enzyme implicated in signal transduction pathways, affecting cell proliferation and differentiation, possibly through the activation of Cyclin D3 [50]. The inositide signaling pathway has been correlated with response to current treatment options for MDS patients yet through different regulation mechanisms in low and high-risk MDS. Namely, PLCβ1 upregulation impairs erythroid differentiation [51], and it has been demonstrated that in low-risk MDS patients responding to erythropoietin, the PI-PLCβ1/cyclin D3 axis is downregulated compared to no responders [52]. On the other hand, in high-risk MDS patients, the combination of azacitidine and valproic acid has demonstrated a potential increased activity by increasing PLCβ [53]. This demonstrates the variable effects of treatment in the same signaling pathway, depending on the MDS subtype (low or high risk). We could assume that HIF-1α signaling inhibition may also have different effects on cultured CD34+ cells from MDS patients with <5% and >5% bone marrow blasts through mechanisms that have not been investigated in the present study but definitely need further elucidation.

## 4. Materials and Methods

### 4.1. Study Population

BΜ aspiration samples, as well as BM biopsies, were collected from 16 newly diagnosed previously untreated MDS patients and seven controls with non-malignant hematologic disorder. MDS subtypes were classified according to WHO 2016 classification [1]. More specifically, the study included the following: 1 MDS with single lineage dysplasia (SLD) patient, 10 MDS with multilineage dysplasia (MLD) patients, 2 MDS with excess blasts 1 (EB1) patients, 2 MDS with excess blasts 2 (EB2) patients, and 1 MDS with isolated del5q patient. The MDS group included 4 females and 12 males with an age ranging from 63 to 90 years (median age 74 years) and the control group 4 females and 3 males with an age ranging from 40 to 83 years (median age 73 years). In the control group, BM aspiration and biopsy were performed for routine diagnostic purposes. The main characteristics of the patients are summarized in Supplementary Table S1.

### 4.2. BM Cell Isolation

Following patient biopsy, whole BM aspirates were tiled on top of a ficoll bilayer column (Histopaque 10771, Histopaque 11191—Sigma Aldrich, St. Louis, MO, USA). After centrifugation, the low-density (upper) and high-density (lower) layers were isolated and washed with PBS. Optical microscopy and flow cytometry allowed characterization of the layers’ cellular contents. The high-density layer contained myeloid lineage cells (morphologic characterization: promyelocytes 1.25%, myelocytes 7.75%, metamyelocytes 5.5%, band cells 5.5%, and neutrophils 80%). The percentages did not differ significantly between the control and the MDS group (*p* > 0.05, Mann–Whitney test). CD34+ were identified in the low-density layer, along with all other mononuclear BM cells. The low-density layer was used for CD34+ cell isolation using magnetic beads (Miltenyi Biotec, Auburn, CA, USA), per the manufacturer’s instructions.

### 4.3. BM Cell Isolation RNA Isolation, cDNA Synthesis and qRT-PCR

CD34+ and myeloid lineage BM cells (high-density layer) were used for mRNA isolation, using a chloroform extraction protocol, and cDNA synthesis was performed (Takara Bio Inc., Kusatsu, Shiga, Japan) qRT-PCR (Kapa Biosystems, Wilmington, MA, USA) for *HIF1A* and *DDIT4/REDD1* was performed as described in Supplementary Table S2.

### 4.4. Flow Cytometry

HIF-1α and REDD1 protein expression, based on median fluorescence intensity (MFI), was determined in BM buffy coat cells, after red blood cells lysis (Cytognos, Salamanca, Spain). Following fixation and permeabilization (IncellDX, San Carlos, CA, USA), intracellular labeling was performed using a mouse anti-human HIF-1α-PE antibody (BioLegend) and polyclonal rabbit anti-human REDD1 antibody (Proteintech). A donkey anti-rabbit immunoglobulin (Ig) G (BioLegend, San Diego, CA, USA) was used as secondary antibody.

HIF-1α and NIM MFI in cultured CD34+ BM cells was assessed on different culture days (namely day 0 and day 9). For HIF-1α, the protocol described above was followed, while the flow cytometric study of NIM expression was performed after direct incubation of the cultured CD34+ BM cells with the IDC labeled NIM.

BM cell populations were distinguished using anti-CD45, CD34, CD117, CD11b, CD14, and CD66b antibodies.

The flow cytometry acquisition was performed on a FACSCanto II flow cytometer.

### 4.5. NIM Dye-Conjugate Synthesis

The NIM dye conjugate used in this work was synthesized as previously described [54]. For each reaction step, the procedure described in the work of Pavlik et al. was followed [54]. Modifications mainly concerned workup or purification methods of the intermediates (Supplementary Table S3).

### 4.6. NIM Staining and Immunofluorescence in BM Biopsies

Right after the acquisition of BM biopsies, specimens were fully immersed in 200 μM NIM solution for 10 min at 37 °C, washed twice in PBS for 1 min, and then immediately introduced to formalin. One BM biopsy incubated with indocyanine green was used as control staining for fluorescent NIM. The specimens were fixed in buffered formalin, decalcified with ethylene diamine tetraacetic acid EDTA/HCl, and embedded in paraffin. Slides with serial sections (7 μm thick) of each sample were studied. Samples were deparaffinized with xylene and rehydrated by incubating in 100% ethanol for 5 min, 96% ethanol for 2 min, 80% ethanol for 2 min, 50% ethanol for 2 min, then in dH_2_O for 1–3 min. Slides were washed in phosphate buffered saline (PBS), immersed in a citrate buffer (10 Mm, pH 6) and incubated in a microwave oven. After cooling, slides were washed in PBS and blocked with blocking solution (2% horse serum) for 45 min at room temperature. DNA was counterstained using 4.6-diamidino-2-phenylindole (DAPI). Immunofluorescence was observed and scanned with a 10× and 40× objective using the Leica TCS-SP8 Confocal Microscope. Full bone marrow biopsies were tile-scanned, and a full bone marrow profile was constructed using 10× objective. Then, 60× close-ups were also obtained from each sample. Fluorescence intensity was calculated correcting for background artifacts for both NIM-ICS and DAPI through meticulous area of interest (ROI) circumscription. Each value obtained was thereafter standardized by division with the negative control NIM-ICS and DAPI immunofluorescence accordingly. To correct for NIM-ICS/DAPI colocalization, standardized NIM-ICS values were divided by the corresponding standardized DAPI fluorescent value. Statistical analysis of the ratios that were calculated was performed and is presented in Figure 2.

### 4.7. Immunofluoresence

Immunofluorescence study was performed in BM cells isolated with ficoll bilayer centrifugation. Cells from high and low density were examined separately. Cells were seeded in lysine-coated glass coverslips and cultured in roswell park memorial institute (RPMI) culture medium supplemented with 2% human serum at 37 °C and 5% CO_2_ for 30 min until they attach. Then, they were fixed with 4% paraformaldehyde solution for 1 h at 4 °C. Nonspecific binding sites were blocked with 5% horse serum in PBS.

For detection of autophagy in specific cell population, samples were stained with a rabbit anti-human LC3B antibody (1:300 dilution; Sigma-Aldrich, St. Louis, MO, USA) along with a mouse anti-human CD34 (Agilent) or mouse anti-human MPO (Agilent, Santa Clara, CA, USA) monoclonal antibody. For further qualitative analysis of autophagy, a monoclonal mouse anti-human LAMP1 antibody (1:200 dilution; OriGene, Rockville, MD, USA) or a monoclonal mouse anti-human TOMM20 antibody (1:200 dilution; Novus Biological, Centenial, CO, USA) were used in combination with anti-LC3B. A polyclonal donkey anti-rabbit and a polyclonal goat anti-mouse antibody were used as secondary antibodies. DNA was counterstained by using DAPI. Appropriate mouse IgGs were used as negative controls. Immunofluorescence was observed and scanned with a 63x/1.4NA objective using the Leica TCS-SP8 Confocal Microscope.

### 4.8. Electron Microscopy

For conventional electron microscopy, the myeloid cells following isolation (high-density layer) were pelletized at 800 g for 5 min. Pellets were fixed with 2.5% glutaraldehyde made up in 0.1 M phosphate buffer (PB) for 1 h at 4 °C. After subsequent buffer washes, pellets were embedded in 4% low-melting agarose in 0.1 M PB. Following solidification, small cubes were cut and post-fixed with 1% osmium tetroxide for 1 h on ice. After washing with the above buffer, the samples were dehydrated in a graded ethanol series and embedded in Epon/Araldite resin mixture. Ultrathin sections were cut with a Diatome diamond knife at a thickness of 65 nm on a Leica EM UC7 ultramicrotome (Leica Microsystems, Vienna, Austria); then, they were mounted onto 300 mesh carbon grids and stained with uranyl acetate and lead citrate. Sections were examined with a Philips 420 Transmission Electron Microscope at an acceleration voltage of 60 kV and photographed with a Megaview G2 CCD camera (Olympus SIS, Münster, Germany).

### 4.9. CD34+ Cell Cultures

Following isolation, CD34+ cells (purity 91% to 98%) from 3 MDS patients with MLD, 3 MDS with EB, and 3 controls were cultured and expanded in 6-well plates (100.000 cells/well). The plates were placed in an incubator at 37 °C, with 5% CO_2_, 85% humidity and 21% O_2_ in the air. Cells were cultured in Iscove’s modified Dulbecco’s medium (IMDM) (Biosera, Metro Manilla, Philippines), supplemented with 10% FBS, penicillin G (5 U/mL), and streptomycin (5 μg/mL). A staged culture protocol was performed for ex vivo expansion and differentiation as a modification of a previously published method [55], which included Stage 1 (days 0–3) and Stage 2 (days 4–16). For each stage, different combinations of growth factors and cytokines were supplemented to the culture media. The combination used were as follows: for Stage 1 (days 0–3), stem cell factor (SCF) 100μg/mL (Peprotech, Rocky Hill, CT, USA), interleukin (IL)-3 10 ng/mL (Peprotech, Rocky Hill, CT, USA), fms like tyrosine kinase 3 ligand (FLT-3L) 100 ng/mL (Peprotech, Rocky Hill, CT, USA), granulocyte colony stimulating factor (G-CSF) 75 ng/mL (Peprotech, Rocky Hill, CT, USA), granulocyte, monocyte colony stimulating factor (GM-CSF) 15 ng/mL (Peprotech, Rocky Hill, CT, USA), and for Stage 2 (days 4–16), SCF 100 ng/mL, FLT-3L 100 ng/mL, and G-CSF 100 ng/mL. Fresh cytokines were replaced every three days. For HIF-1 inhibition, CD34+cultures were performed under the same conditions described above with the addition of 10 μM HIF-1α inhibitor (methyl 3-[[2-[4-(2-adamantyl)phenoxy]acetyl]amino]-4-hydroxybenzoate, Santa Cruz Biotechnology) from day 0. Cell numbers and morphological characteristics were determined every three days using an automated cell counting system (Coulter, Brea, CA, USA) and cell cytospins stained with May–Grunwald–Giemsa reagents (Merck, Keniworth, NJ, USA) respectively.

### 4.10. Statistics

Statistical analyses were performed using Mann–Whitney tests non-parametric tests. All data were analyzed using GraphPad Prism v8 software (San Diego, CA USA, www.graphpad.com, accessed on 10 April 2021). Data are presented as mean ± SD. Differences considered statistically significant at *p* < 0.05 level.

## 5. Conclusions

In this study, we experimentally test the hypothesis of an aberrant, non-canonical, oxygen-independent HIF-1 activity as a prominent feature of the MDS pathobiology along with its potential as a therapeutic target. We conclude that although HIF-1α is aberrantly expressed and non-canonically regulated in all MDS irrespective of intra BM blast percentage, at least when referring to the myeloid lineage, its expression is most likely the result of a global HSCs interruption with variable contribution in the disease establishment and evolution. Its therapeutic targeting is probably of limited potential and mainly confined to MDS with low blast percentage. Overall, our work suggests that the MDS BM is a holistically disrupted nest of faulty CD34+ HSCs, which start differentiating surrounded by a dysfunctional microenvironment that eventually dictates their premature death or malignant transformation. We show that pseudohypoxia most likely due to mitochondrial dysfunction is evidently present in all MDS subtypes. While MDS with excess blasts (≥5%) demonstrate a pseudohypoxic phenotype caused by faulty respiration, as featured by their pathologic mitochondrial morphology, benign MDS with <5% blasts acquire the same pseudohypoxic phenotype due to the marked degradation of their dysfunctional mitochondria. This mitochondrial dysfunction induces a secondary HIF-1α stabilization and sequentially causes NIM-IDC accumulation.

## Supplementary Materials

The following are available online at https://www.mdpi.com/article/10.3390/ijms22084099/s1, Table S1: MDS patient characteristics, Table S2: Primer sequences for qRT PCR, Table S3: Synthesis of NIM dye conjugate, Figure S1: (A) HIF1A and REDD1 mRNA expression in CD34+ and myeloid cells of MDS patients (n = 16) and controls (n = 7) (B) REDD1 mRNA expression in in vitro cultures of CD34+ cells after 16 days of differentiation. Figure S2: Immunofluorescence for CD34 and LC3B in one control and one MDS sample, Figure S3: Electron microscopy of normal differentiating myeloid lineage (control sample) at the myelocyte–metamyelocyte stage, Figure S4: Electron microscopy of mature MDS myeloid lineage showing marked autophagy, mitophagy, and eventual autophagic cell death (autosis) with cytoplasmic abundance of autophagosomes and perinuclear membrane convolution, Figure S5: Immunofluorescence of LC3B and LAMP-1 in myeloid cells of control (CTRL-A) and MDS (B and C) patients.

## References

[1] Arber D.A., Orazi A., Hasserjian R., Thiele J., Borowitz M.J., Le Beau M.M., Bloomfield C.D., Cazzola M., Vardiman J.W. (2016). The 2016 revision to the World Health Organization classification of myeloid neoplasms and acute leukemia. Blood.

[2] Hofmann W.K., Koeffler H.P. (2005). Myelodysplastic syndrome. Annu. Rev. Med..

[3] Hsu J., Reilly A., Hayes B.J., Clough C.A., Konnick E.Q., Torok-Storb B., Gulsuner S., Wu D., Becker P.S., Keel S.B. (2019). Reprogramming identifies functionally distinct stages of clonal evolution in myelodysplastic syndromes. Blood.

[4] Mossner M., Jann J.C., Wittig J., Nolte F., Fey S., Nowak V., Obländer J., Pressler J., Palme I., Xanthopoulos C. (2016). Mutational hierarchies in myelodysplastic syndromes dynamically adapt and evolve upon therapy response and failure. Blood.

[5] Papaemmanuil E., Gerstung M., Malcovati L., Tauro S., Gundem G., Van Loo P., Yoon C.J., Ellis P., Wedge D.C., Pellagatti A. (2013). Clinical and biological implications of driver mutations in myelodysplastic syndromes. Blood.

[6] Steensma D.P., Bejar R., Jaiswal S., Lindsley R.C., Sekeres M.A., Hasserjian R.P., Ebert B.L. (2015). Clonal hematopoiesis of indeterminate potential and its distinction from myelodysplastic syndromes. Blood.

[7] Acuna-Hidalgo R., Sengul H., Steehouwer M., van de Vorst M., Vermeulen S.H., Kiemeney L., Veltman J.A., Gilissen C., Hoischen A. (2017). Ultra-sensitive Sequencing Identifies High Prevalence of Clonal Hematopoiesis-Associated Mutations throughout Adult Life. Am. J. Hum. Genet..

[8] Masala E., Valencia-Martinez A., Pillozzi S., Rondelli T., Brogi A., Sanna A., Gozzini A., Arcangeli A., Sbarba P.D., Santini V. (2018). Severe hypoxia selects hematopoietic progenitors with stem cell potential from primary Myelodysplastic syndrome bone marrow cell cultures. Oncotarget.

[9] Thompson J.E., Conlon J.P., Yang X., Sanchez P.V., Carroll M. (2007). Enhanced growth of myelodysplastic colonies in hypoxic conditions. Exp. Hematol..

[10] Ito K., Bonora M., Ito K. (2019). Metabolism as master of hematopoietic stem cell fate. Int. J. Hematol..

[11] Kohli L., Passegué E. (2014). Surviving change: The metabolic journey of hematopoietic stem cells. Trends Cell Biol..

[12] Suda T., Takubo K., Semenza G.L. (2011). Metabolic regulation of hematopoietic stem cells in the hypoxic niche. Cell Stem. Cell.

[13] Zhang C.C., Sadek H.A. (2014). Hypoxia and metabolic properties of hematopoietic stem cells. Antioxid. Redox Signal..

[14] Takubo K., Goda N., Yamada W., Iriuchishima H., Ikeda E., Kubota Y., Shima H., Johnson R.S., Hirao A., Suematsu M. (2010). Regulation of the HIF-1alpha level is essential for hematopoietic stem cells. Cell Stem. Cell.

[15] Wang G.L., Jiang B.H., Rue E.A., Semenza G.L. (1995). Hypoxia-inducible factor 1 is a basic-helix-loop-helix-PAS heterodimer regulated by cellular O2 tension. Proc. Natl. Acad. Sci. USA.

[16] Kim J.W., Tchernyshyov I., Semenza G.L., Dang C.V. (2006). HIF-1-mediated expression of pyruvate dehydrogenase kinase: A metabolic switch required for cellular adaptation to hypoxia. Cell Metab..

[17] Liu W., Shen S.M., Zhao X.Y., Chen G.Q. (2012). Targeted genes and interacting proteins of hypoxia inducible factor-1. Int. J. Biochem. Mol. Biol..

[18] Lu H., Forbes R.A., Verma A. (2002). Hypoxia-inducible factor 1 activation by aerobic glycolysis implicates the Warburg effect in carcinogenesis. J. Biol. Chem..

[19] Semenza G.L. (2007). Oxygen-dependent regulation of mitochondrial respiration by hypoxia-inducible factor 1. Biochem. J..

[20] Zhang H., Bosch-Marce M., Shimoda L.A., Tan Y.S., Baek J.H., Wesley J.B., Gonzalez F.J., Semenza G.L. (2008). Mitochondrial autophagy is an HIF-1-dependent adaptive metabolic response to hypoxia. J. Biol. Chem..

[21] Yang C., Suda T. (2018). Hyperactivated mitophagy in hematopoietic stem cells. Nat. Immunol..

[22] Shoshani T., Faerman A., Mett I., Zelin E., Tenne T., Gorodin S., Moshel Y., Elbaz S., Budanov A., Chajut A. (2002). Identification of a novel hypoxia-inducible factor 1-responsive gene, RTP801, involved in apoptosis. Mol. Cell. Biol..

[23] Hayashi Y., Yokota A., Harada H., Huang G. (2019). Hypoxia/pseudohypoxia-mediated activation of hypoxia-inducible factor-1α in cancer. Cancer Sci..

[24] Iommarini L., Porcelli A.M., Gasparre G., Kurelac I. (2017). Non-Canonical Mechanisms Regulating Hypoxia-Inducible Factor 1 Alpha in Cancer. Front. Oncol..

[25] Klionsky D.J., Abdelmohsen K., Abe A., Abedin M.J., Abeliovich H., Acevedo Arozena A., Adachi H., Adams C.M., Adams P.D., Adeli K. (2016). Guidelines for the use and interpretation of assays for monitoring autophagy (3rd edition). Autophagy.

[26] Kishi-Itakura C., Buss F. (2018). The Use of Correlative Light-Electron Microscopy (CLEM) to Study PINK1/Parkin-Mediated Mitophagy. Methods Mol. Biol..

[27] Gustafson M.P., Lin Y., Maas M.L., Van Keulen V.P., Johnston P.B., Peikert T., Gastineau D.A., Dietz A.B. (2015). A method for identification and analysis of non-overlapping myeloid immunophenotypes in humans. PLoS ONE.

[28] Nunn A., Linder K., Strauss H.W. (1995). Nitroimidazoles and imaging hypoxia. Eur. J. Nucl. Med..

[29] Takanuki K., Igarashi T., Hata K., Hori H., Shibata T., Kitagawa H., Satoh T., Inayama S. (1988). Glutathione depletion by 2-nitroimidazole-1-acetohydroxamic acid as a radiosensitizer in hypoxic rat hepatocytes. Biochem. Int..

[30] Liu K., Zhu H.L. (2011). Nitroimidazoles as anti-tumor agents. Anticancer Agents Med. Chem..

[31] Krohn K.A., Link J.M., Mason R.P. (2008). Molecular imaging of hypoxia. J. Nucl. Med..

[32] Westrate L.M., Drocco J.A., Martin K.R., Hlavacek W.S., MacKeigan J.P. (2014). Mitochondrial morphological features are associated with fission and fusion events. PLoS ONE.

[33] Michaela F., Emmanuel G. (2008). Apoptotic pathways to death in myelodysplastic syndromes. Haematologica.

[34] Poulaki A., Katsila T., Stergiou I.E., Giannouli S., Gόmez-Tamayo J.C., Piperaki E.T., Kambas K., Dimitrakopoulou A., Patrinos G.P., Tzioufas A.G. (2020). Bioenergetic Profiling of the Differentiating Human MDS Myeloid Lineage with Low and High Bone Marrow Blast Counts. Cancers.

[35] Twig G., Hyde B., Shirihai O.S. (2008). Mitochondrial fusion, fission and autophagy as a quality control axis: The bioenergetic view. Biochim. Biophys. Acta.

[36] Twig G., Elorza A., Molina A.J.A., Mohamed H., Wikstrom J.D., Walzer G., Stiles L., Haigh S.E., Katz S., Las G. (2008). Fission and selective fusion govern mitochondrial segregation and elimination by autophagy. EMBO J..

[37] Liesa M., Shirihai O.S. (2013). Mitochondrial dynamics in the regulation of nutrient utilization and energy expenditure. Cell Metab..

[38] Brugarolas J., Lei K., Hurley R.L., Manning B.D., Reiling J.H., Hafen E., Witters L.A., Ellisen L.W., Kaelin W.G. (2004). Regulation of mTOR function in response to hypoxia by REDD1 and the TSC1/TSC2 tumor suppressor complex. Genes Dev..

[39] Bellot G., Garcia-Medina R., Gounon P., Chiche J., Roux D., Pouysségur J., Mazure N.M. (2009). Hypoxia-induced autophagy is mediated through hypoxia-inducible factor induction of BNIP3 and BNIP3L via their BH3 domains. Mol. Cell Biol.

[40] Riffelmacher T., Simon A.-K. (2017). Mechanistic roles of autophagy in hematopoietic differentiation. FEBS J..

[41] Riffelmacher T., Clarke A., Richter F.C., Stranks A., Pandey S., Danielli S., Hublitz P., Yu Z., Johnson E., Schwerd T. (2017). Autophagy-Dependent Generation of Free Fatty Acids Is Critical for Normal Neutrophil Differentiation. Immunity.

[42] Liu Y., Levine B. (2015). Autosis and autophagic cell death: The dark side of autophagy. Cell Death Differ..

[43] Liu Y., Shoji-Kawata S., Sumpter R.M., Wei Y., Ginet V., Zhang L., Posner B., Tran K.A., Green D.R., Xavier R.J. (2013). Autosis is a Na+,K+-ATPase-regulated form of cell death triggered by autophagy-inducing peptides, starvation, and hypoxia-ischemia. Proc. Natl. Acad. Sci. USA.

[44] Hayashi Y., Zhang Y., Yokota A., Yan X., Liu J., Choi K., Li B., Sashida G., Peng Y., Xu Z. (2018). Pathobiological Pseudohypoxia as a Putative Mechanism Underlying Myelodysplastic Syndromes. Cancer Discov..

[45] Agani F., Jiang B.H. (2013). Oxygen-independent regulation of HIF-1: Novel involvement of PI3K/AKT/mTOR pathway in cancer. Curr Cancer Drug Targets.

[46] Braccini L., Ciraolo E., Martini M., Pirali T., Germena G., Rolfo K., Hirsch E. (2012). PI3K keeps the balance between metabolism and cancer. Adv. Biol. Regul..

[47] Nepstad I., Hatfield K.J., Grønningsæter I.S., Reikvam H. (2020). The PI3K-Akt-mTOR Signaling Pathway in Human Acute Myeloid Leukemia (AML) Cells. Int. J. Mol. Sci..

[48] Nyåkern M., Tazzari P.L., Finelli C., Bosi C., Follo M.Y., Grafone T., Piccaluga P.P., Martinelli G., Cocco L., Martelli A.M. (2006). Frequent elevation of Akt kinase phosphorylation in blood marrow and peripheral blood mononuclear cells from high-risk myelodysplastic syndrome patients. Leukemia.

[49] Follo M.Y., Mongiorgi S., Bosi C., Cappellini A., Finelli C., Chiarini F., Papa V., Libra M., Martinelli G., Cocco L. (2007). The Akt/Mammalian Target of Rapamycin Signal Transduction Pathway Is Activated in High-Risk Myelodysplastic Syndromes and Influences Cell Survival and Proliferation. Cancer Res..

[50] Faenza I., Matteucci A., Manzoli L., Billi A.M., Aluigi M., Peruzzi D., Vitale M., Castorina S., Suh P.G., Cocco L. (2000). A role for nuclear phospholipase Cbeta 1 in cell cycle control. J. Biol. Chem..

[51] Manzoli L., Billi A.M., Gilmour R.S., Martelli A.M., Matteucci A., Rubbini S., Weber G., Cocco L. (1995). Phosphoinositide signaling in nuclei of Friend cells: Tiazofurin down-regulates phospholipase C beta 1. Cancer Res..

[52] Follo M.Y., Mongiorgi S., Clissa C., Paolini S., Martinelli G., Martelli A.M., Fioravanti G., Manzoli L., Finelli C., Cocco L. (2012). Activation of nuclear inositide signalling pathways during erythropoietin therapy in low-risk MDS patients. Leukemia.

[53] Follo M.Y., Finelli C., Mongiorgi S., Clissa C., Chiarini F., Ramazzotti G., Paolini S., Martinelli G., Martelli A.M., Cocco L. (2011). Synergistic induction of PI-PLCβ1 signaling by azacitidine and valproic acid in high-risk myelodysplastic syndromes. Leukemia.

[54] Pavlik C., Biswal N.C., Gaenzler F.C., Morton M.D., Kuhn L.T., Claffey K.P., Zhu Q., Smith M.B. (2011). Synthesis and fluorescent characteristics of imidazole–indocyanine green conjugates. Dyes Pigment..

[55] Jie Z., Zhang Y., Wang C., Shen B., Guan X., Ren Z., Ding X., Dai W., Jiang Y. (2017). Large-scale ex vivo generation of human neutrophils from cord blood CD34+ cells. PLoS ONE.

