# Supplementary material for: Exploiting the Role of Hypoxia-Inducible Factor 1 and Pseudohypoxia in the Myelodysplastic Syndrome Pathophysiology
Source: Int J Mol Sci. 2021 Apr 15;22(8):4099. doi: 10.3390/ijms22084099 (PMC8071466; doi:10.3390/ijms22084099)
Supplement: Supplementary file 1 [file ijms-22-04099-s001.zip › ijms-1168069-SI.pdf]

Patient ID	Age (years)	Gender	MDS subtype	IPSS-R	Hb (g/dl)	WBC (K/μl)	Neutrophils (K/μl)	Platelets (K/μl)	BM Cytogenetics	Peripheral Blood Blasts (% of nucleated cells)†	BM Blasts (% of nucleated cells)††	BM cellularity*	Transfusion Dependence
1	81	Female	MLD	1 (very low)	11,3	4,74	2,70	139	46,XX[20]	0	2	Normocellular	No
2	83	Male	MLD	1 (very low)	13,0	3,53	1,90	142	46,XY[20]	0	2	Hypocellular	No
3	78	Male	MLD	2,5 (low)	6,6	3,25	1,25	116	46,XY[20]	0	3	Hypocellular	Yes
4	74	Female	Isolated del(5q)	2 (low)	8,5	2,77	1,67	386	46,XX,del(5q)[20]	0	1	Normocellular	
5	82	Female	MLD	1 (very low)	10,6	4,50	3,15	177	46,XX[20]	0	2	Normocellular	No
6	77	Male	MLD	1 (very low)	9,3	4.90	2,74	324	46,X,-Y[20]	2	3	Normocellular	Yes
7	80	Male	SLD	3 (low)	8,8	8,18	5,64	520	46,XY[20]	0	0	Hypercellular	Yes
8	78	Male	MLD	3 (low)	9,5	2,38	1,67	148	46,XY[20]	0	4	Normocellular	Yes
9	77	Male	EB 1	6 (high)	9,4	1,83	0,60	56	46,XY, del(5)(q11.2q33), del(20)(q11.2~13.1)[18]/46,XY[1]	1	8	Normocellular	
10	85	Male	MLD	3,5 (intermediate)	5,6	3.48	2,50	131	47,XY,+8[20]/46,XY[5]	0	0	Normocellular	Yes
11	63	Male	MLD	2 (low)	10,7	3,16	0,90	274	46,XY, inv(9)(p12q13)[12]	0	0	Hypocellular	No
12	79	Female	EB 2	6 (high)	9,0	4,47	2,00	103	47,XX,+8[20]	5	15	Hypercellular	No
13	90	Male	EB 1	7,5 (very high)	4,7	9,32	6,90	184	46,XY, del(7)(q21)[6]/45,XY?add(1)(p36),add(2)(q37),del(7)(q21),-20[15]	2	8	Normocellular	Yes
14	75	Male	EB 2	7 (very high)	8,6	1,90	0,50	58	47,XY,+8[23]/46,XY[2]	3	15	Hypercellular	Yes
15	81	Male	MLD	3(low)	8,0	1,78	0,50	145	47,XY,+8[23]/46,XY[2]	0	0	Hypercellular	No
16	81	Male	MLD	2(low)	8,9	2,11	1,0	120	46,XY, del(20)(q11.2)[30]	0	1	Hypercellular	No

Supplementary Table 1. MDS Patients characteristics.

IPSS-R: Revised International Prognostic Scoring System, Hb: hemoglobin, WBC: white blood cells, BM: bone marrow, MLD: multilineage dysplasia, SLD: single lineage dysplasia, EB: excess blasts

† assessed by morphology in peripheral blood smears,†† assessed by morphology of bone marrow smears, *assessed in bone marrow biopsy

Gene	Primer	Sequence of primers	RT-PCR conditions
DDIT4	Forward<br>Reverse	5’ -GAGGAAGACACGGCTTAC 3’<br>5’ -GCATCAGGTTGGCACAC 3’	Annealing/Elongation<br>52oC 40 min
HIF1A	Forward<br>Reverse	5’ -CAGGACACAGATTTAGACTTGGAGA 3’<br>5’ -AGTGGTAGTGGTGGCATTAGC 3’	Annealing/Elongation<br>52oC 40 min
GAPDH	Forward<br>Reverse	5’ -GGGAAGCTTGTCATCAATGG 3’<br>5’ -CATCGCCCCACTTGATTTTG 3’	Annealing/Elongation<br>52oC 40 min

Supplementary Table 2. Primer sequences for qRT PCR

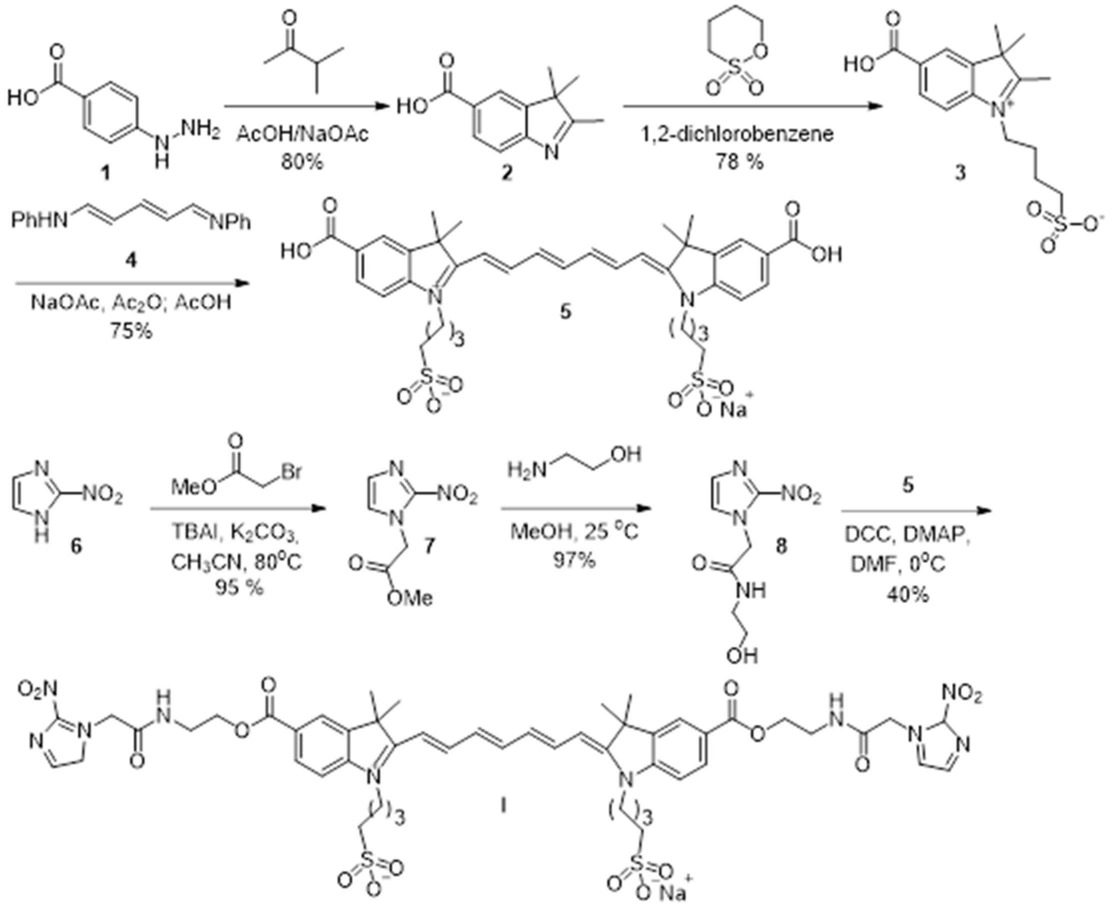

Supplementary Table 3. Synthesis of NIM dye-conjugate - developed by Pavlik et al.[1] Modifications mainly concerned workup or purification methods of the intermediates (Supplementary Table 3). In particular, in the synthesis of 2,3,3-trimethyl-3H-indole-5-carboxylic acid 2, the resulted residue was participated between CH2Cl2 and H2O, the two phases were separated, the aqueous phase was washed with CH2Cl2 and the combined organic phases were dried over Na2SO4 and evaporated in vacuo. Purification of the residue was performed by flash column chromatography (SiO2, CHCl3/CH3OH 95:5) and 2 was obtained as a bright orange solid. With the use of purified 2 in the synthesis of 5-carboxy-1-(d-sulfobutyl)-2,3,3-trimethyl-3H-indolium betaine 3, the reaction was completed in 5 h of reflux and 3 was afforded as an orange solid (Supplemental Table 3). Methyl 2-(2-nitro-1H-imidazol-1-yl)acetate 7 was purified by a short pad flash column chromatography (SiO2, CHCl3/MeOH 95:5 to 9:1) and it was afforded as a white solid .

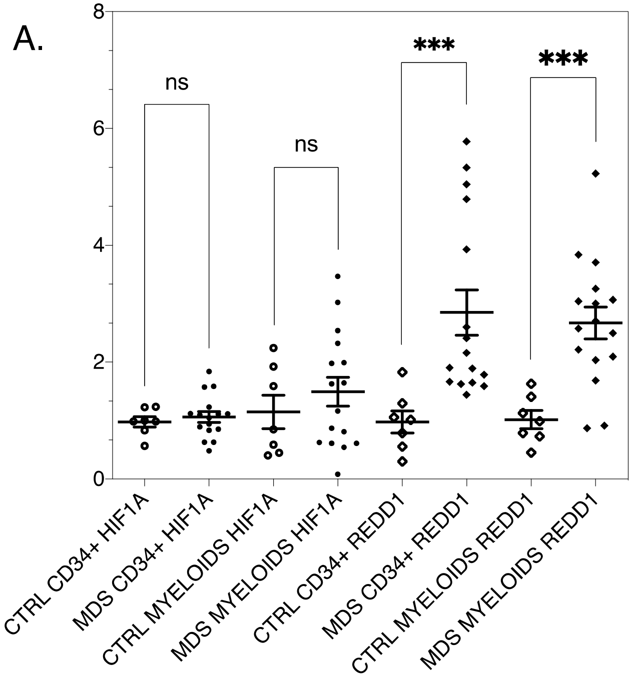

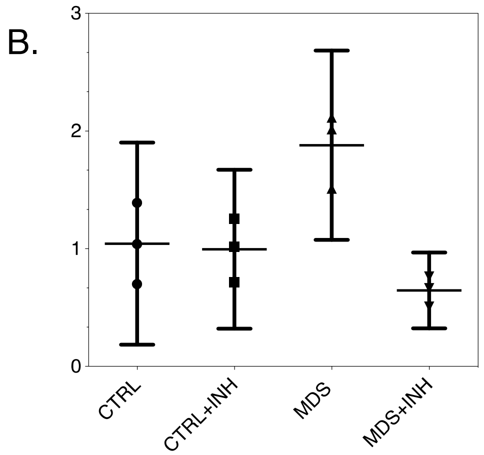

CTRL	CTRL+INH	MDS	MDS+INH
1,038859	1,257013	2,114036	0,6649025
1,389918	1,01748	1,510473	0,5077861
0,6997929	0,7144971	2,014036	0,7649025

Supplementary Figure 1: (A) HIF1A and REDD1 mRNA expression in CD34+ and myeloid cells of MDS patients (n=16) and controls (n=7). ns: non-significant, sig.: significant. Mann-Whitney Pvalue>0.05 or <0.05 accordingly. (B) REDD1 mRNA expression in in vitro cultures of CD34+ cells after 16 days of differentiation. Data from three independent experiments presented as along with mean±SD. Table shows graph displayed numbers

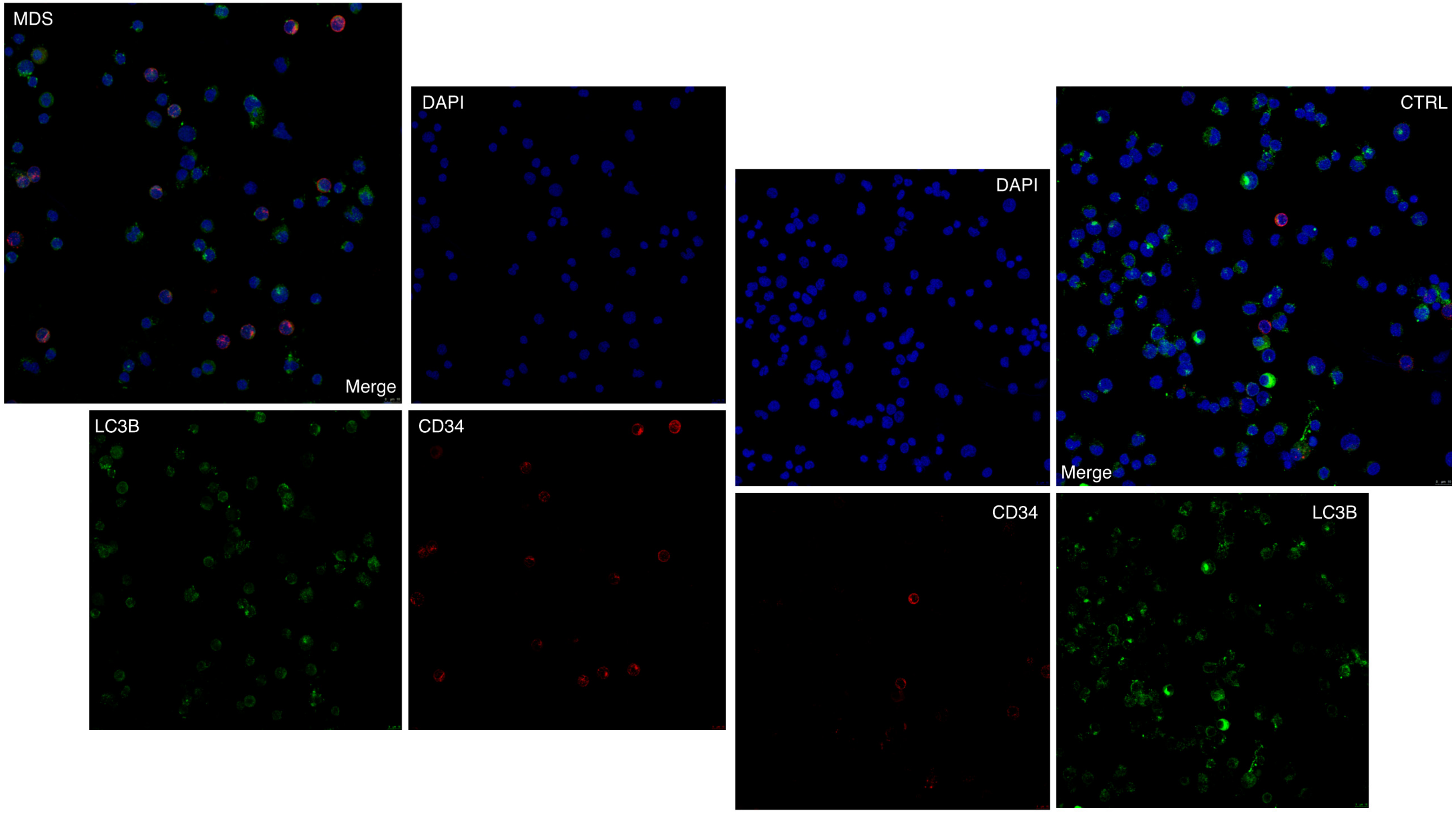

Supplementary Figure 2: Immunofluorescence for CD34 and LC3B in one control (CTRL-A) and one MDS (B) sample. Red: MPO, Green: LC3B, Blue: DAPI. Objective 63x/1.4NA. One out of four independent experiments. Cells shown represent the mononuclear-low density layer following bone marrow aspirate ficoll bilayer isolation. Aside from CD34+ percentage no differences are observed in LC3b immunofluorescence. Patient belonged to the high risk group, >5% intra bone marrow blasts as also evident from CD34 immunofluorescence. LC3B: Microtubule-associated proteins 1A/1B light chain 3B.

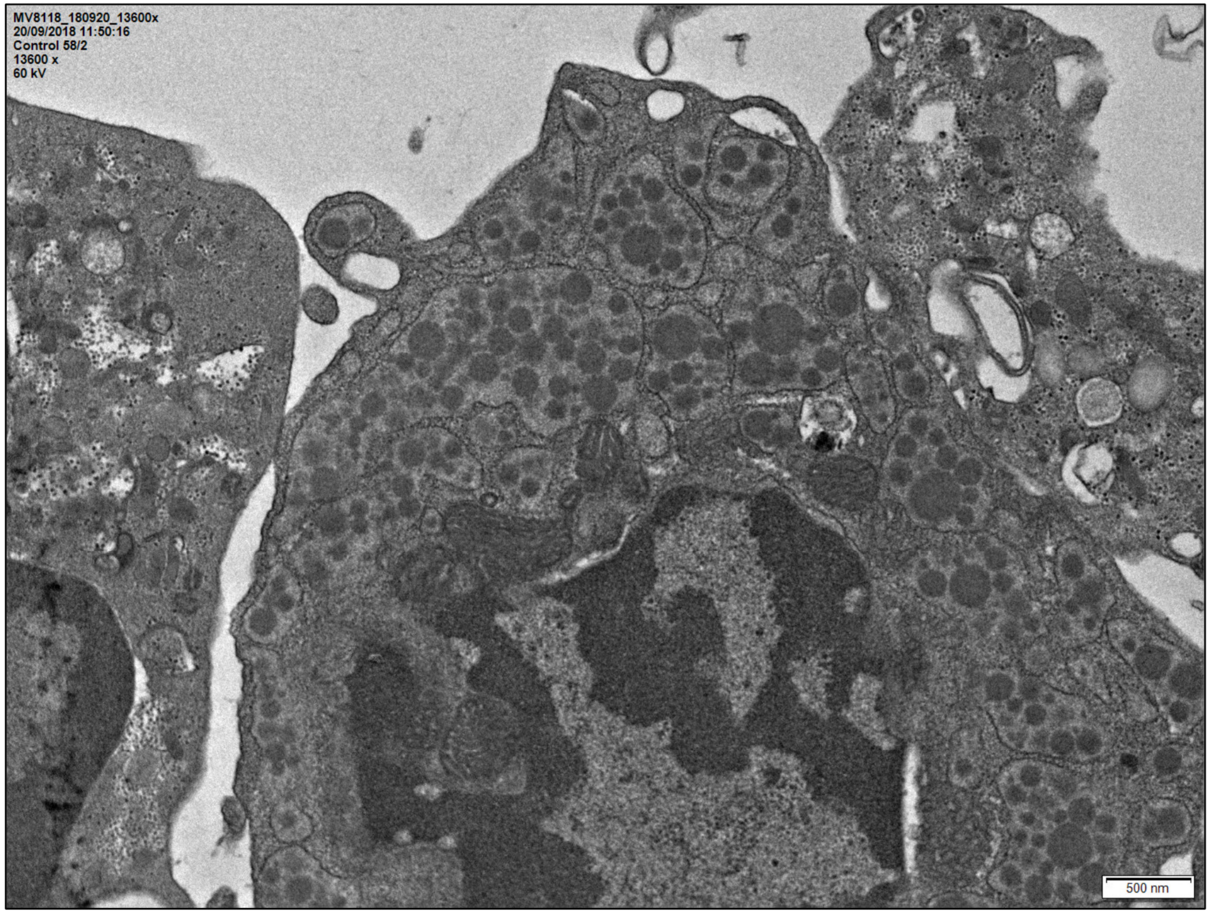

Supplementary Figure 3: Electron microscopy of normal differentiating myeloid lineage (control sample) at the myelocyte-metamyelocyte stage. Normal mitochondria with physiologic round appearance and functional cristae. Scale bars and magnifications are shown on the upper left corner of each picture.

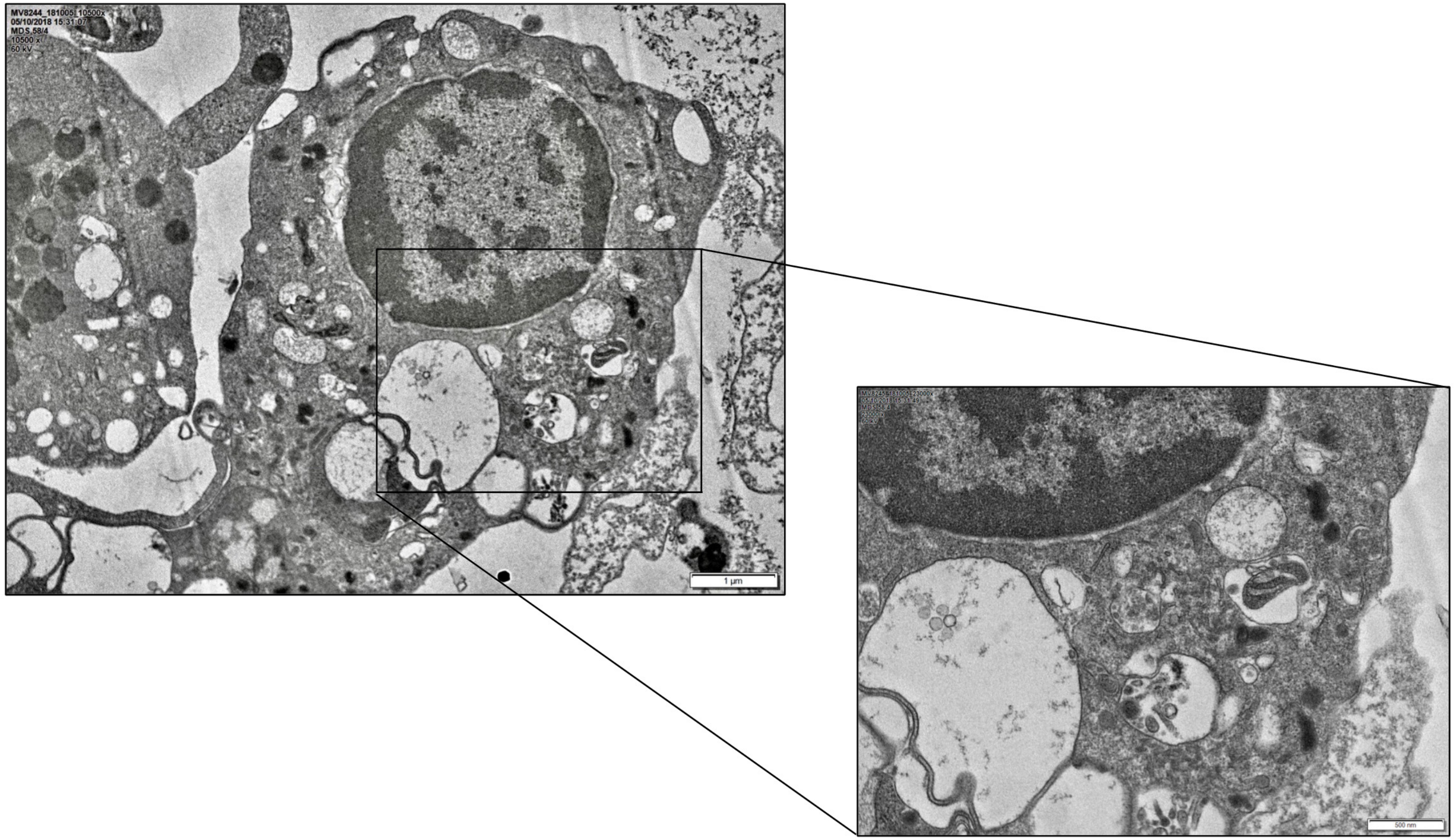

Supplementary Figure 4: Electron microscopy of mature MDS myeloid lineage showing marked autophagy, mitophagy and eventual autophagic cell death (autosis) with cytoplasmic abundance of autophagosomes and perinuclear membrane convolution. Large elongated abnormal mitochondria are also evident. One of six independent experiments. Scale bars and magnifications are shown on the upper left corner of each picture. Note patients showing autosis are almost exclusively of the high risk MDS subgroup, namely with >5% intra bone marrow blasts.

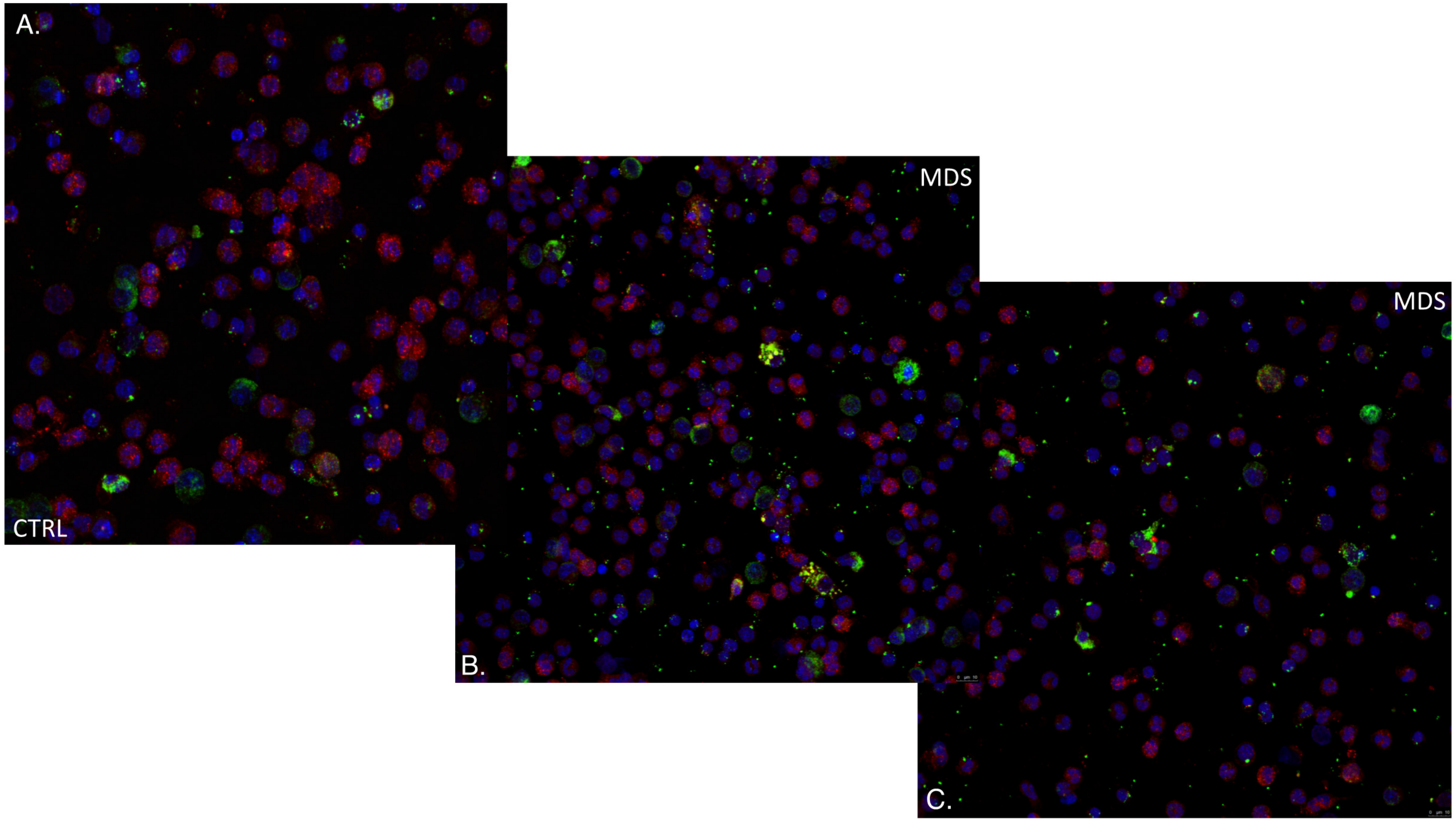

Supplementary Figure 5: Immunofluorescence of LC3B and LAMP-1 in myeloid cells of control (CTRL-A) and MDS (B and C) patients. Objective 63x/1.4NA. Red: LAMP1, Green: LC3B, Blue: DAPI. LC3B: Microtubule-associated proteins 1A/1B light chain 3B, LAMP-1: Lysosomal-associated membrane protein 1.Only the merged files are shown.

References

1. Pavlik C, Biswal NC, Gaenzler FC, Morton MD, Kuhn LT, Claffey KP, et al. Synthesis and fluorescent characteristics of imidazole–indocyanine green conjugates. Dyes and Pigments. 2011 2011/04/01/;89(1):9-15.
